# A Review of Secondary Flow in Inertial Microfluidics

**DOI:** 10.3390/mi11050461

**Published:** 2020-04-28

**Authors:** Qianbin Zhao, Dan Yuan, Jun Zhang, Weihua Li

**Affiliations:** 1 School of Mechanical, Materials, Mechatronic and Biomedical Engineering, University of Wollongong, Wollongong, NSW 2522, Australia; qz260@uowmail.edu.au; 2 Department of Chemistry, University of Tokyo, Tokyo 113-0033, Japan; danyuan@chem.s.u-tokyo.ac.jp; 3 Queensland Micro and Nanotechnology Centre, Griffith University, Brisbane, QLD 4111, Australia

**Keywords:** secondary flow, Dean flow, inertial microfluidics, particle manipulation and separation

## Abstract

Inertial microfluidic technology, which can manipulate the target particle entirely relying on the microchannel characteristic geometry and intrinsic hydrodynamic effect, has attracted great attention due to its fascinating advantages of high throughput, simplicity, high resolution and low cost. As a passive microfluidic technology, inertial microfluidics can precisely focus, separate, mix or trap target particles in a continuous and high-flow-speed manner without any extra external force field. Therefore, it is promising and has great potential for a wide range of industrial, biomedical and clinical applications. In the regime of inertial microfluidics, particle migration due to inertial effects forms multiple equilibrium positions in straight channels. However, this is not promising for particle detection and separation. Secondary flow, which is a relatively minor flow perpendicular to the primary flow, may reduce the number of equilibrium positions as well as modify the location of particles focusing within channel cross sections by applying an additional hydrodynamic drag. For secondary flow, the pattern and magnitude can be controlled by the well-designed channel structure, such as curvature or disturbance obstacle. The magnitude and form of generated secondary flow are greatly dependent on the disturbing microstructure. Therefore, many inventive and delicate applications of secondary flow in inertial microfluidics have been reported. In this review, we comprehensively summarize the usage of the secondary flow in inertial microfluidics.

## 1. Introduction

Microfluidic technology is defined as the precise manipulation of very tiny quantities of fluids (from 10^−9^ to 10^−18^ L) by leveraging the delicate channel with the dimension of tens to hundreds of micrometers [1]. In the past decades, the development of microfluidic technologies is booming, with applications in bioanalysis, chemical synthesis, medical chemistry, cell biological research, etc. [1]. As a rapidly developing technology, there are many fascinating advantages of microfluidics compared with the traditional macroscale technology, including (1) less sample and reagent amounts required; (2) reduction of analytic time; (3) high detection sensitivity; (4) low cost; (5) small footprint and (6) good automation and integration characteristics for less risk of human intervention, etc. [2].

Owing to the capability of manipulating fluids at the order of microliter level, microfluidics is expected to be the revolutionary technology in fields of biomedicine and clinical diagnostics. Precise particle manipulation is an essential ability in most biological assays and medical diagnostics [3]. Particles (e.g., cells) can be orderly manipulated based on differences in their biological and physical characteristics, such as size, shape, magnetism, dielectric property, density, etc. [3,4,5]. 

Many microfluidic technologies of particle manipulation have been developed. According to the manipulation principle, microfluidic particle-manipulation technology can be classified as either passive or active methods. The active methods refer to the ones taking advantage of the external field for its functionality. In contrast, the passive methods merely rely on the channel geometry and intrinsic hydrodynamic effects for effective particle manipulation. The high-sensitivity acoustophoresis (AP) [6], dielectrophoresis (DEP) [7], magnetophoresis (MP) [8], optical force [9], electrokinetics [10] and their combination [11] are typically employed by the active microfluidic particle-manipulation methods. In general, the active particle manipulation can provide and contribute to a more precise particle movement, but the external functional field restricts the throughput as they require particles to be exerted for a sufficient period. Moreover, bulky auxiliary equipment complicates the whole system and complex fabrication process increases the cost of device. On the other hand, the passive microfluidic methods, such as deterministic lateral displacement (DLD) [12,13], pinched-flow fractionation (PFF) [14], hydrophoresis [15,16] and inertial microfluidics [4,17,18], are normally more convenient to operate and have relatively higher throughput.

In microfluidics, an extra lateral force that is perpendicular to the primary flow direction is always needed to move the target particles or fluid to the desired position. This lateral force can be: (1) an electrical [7], acoustic [6] or magnetic [8] force, originating from an external power source; or (2) a hydrodynamic effect due to intrinsic fluid properties and fluid lateral movement, such as inertial migration, viscoelastic focusing and secondary flow [4,19]. For the microfluidic technology that employs external active force fields, inertial migration, and viscoelastic focusing, there have been technical review articles that specifically discussed each technology [4,17,20,21]. 

Secondary flow, which is the relatively minor flow perpendicular to the primary flow, can apply an extra force on the particles and displace their positions. This hydrodynamic force is normally associated with the inertial flow regime, serving as one of the most important effects for implementation of particle manipulation. Secondary flow has been extensively employed for fluid and particle manipulation, such as mixing, trapping, focusing and separation [3,17,22]. However, to the best of the authors’ knowledge, so far, there has been no technical review to summarize and discuss specifically the mechanism and usage of secondary flow on the manipulation of fluid and particles in microfluidics. 

**Figure 1:**
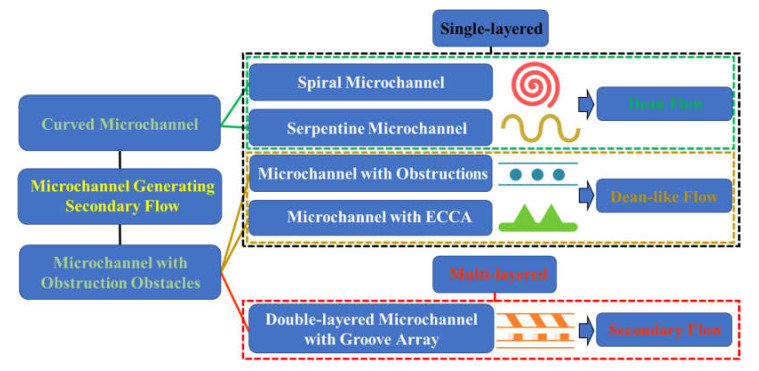
Category illustration of microchannels using secondary flow. There are five typical types of microchannels summarized in our work, which can generate secondary flow through the curved channel or obstruction obstacle. They are the spiral microchannel, serpentine microchannel, microchannel with expansion-contraction cavity array (ECCA), microchannel with obstructions and double-layered microchannel with groove array. The single-layered microchannel generates the specific type of secondary flow, Dean flow and Dean-like flow, to control particle movement, whereas the multilayered microchannel induces complicated irregular secondary flow.

Therefore, in this review, we discuss the mechanism of secondary flow in various structured channels and summarize their applications on the fluid mixing and particle manipulation. Based on the channel feature, we categorize the channel structures as a spiral or serpentine channel, a channel with obstructions or with expansion-contraction cavity arrays (ECCA), or a multilayer channel with top/bottom groove array, Figure 1. Then, we discuss the mechanism and characteristics of secondary flow in each channel structure. And it is found that Dean flow, Dean-like flow and irregular secondary flow are correspondingly generated according to the disturbing microstructure. After that, we summarize their biomedical applications. Finally, we point out some perspectives on the future development of secondary flow usage in inertial microfluidics. This review article is expected to provide a deep insight into secondary flow and its up-to-date biomedical applications. 

## 2. Hydrodynamic Force in Microchannel

Inertial microfluidics is one of the most popular passive technologies relying on natural inertial effects at high flow speed. Originally, the inertia of fluid and particles in the microfluidic system was ignored. There used to be a misconception that the flow condition of laminar flow can be equated to Stokes flow [4]. However, the inconsistent results between the experiments and this approximation always imply the unreasonable aspect, especially with the increase of *Re* ($Re=\rho_{f}UH/\mu$ $\rho_{f}$: fluid density; U: maximum velocity; H: hydraulic diameter of microchannel; μ: dynamic viscosity). Therefore, an increasing number of research works began to focus on the intermediate flow condition (*Re* is from ~1 to ~100) [23,24].

Early in the 1960s, it was found that particles which were disorderly dispersed at the entrance of a straight channel would gradually migrate laterally to several equilibrium positions without any external intervention after travelling a long enough distance [25,26]. This intriguing phenomenon which is referred to the inertial migration has been comprehensively investigated and widely recognized by the counteraction of two dominated forces in the inertial regime, the shear gradient lift force $F_{\mathrm{Ls}}$ ($F_{\mathrm{Ls}}\propto \rho_{f}U^{2}a^{3}/H$, a : particle diameter) and the wall lift force $F_{\mathrm{Lw}}$ ($F_{\mathrm{Lw}}\propto \rho_{f}U^{2}a^{6}/H^{4}$) [27,28]. Wall lift force is induced by the interaction of suspending particles and adjacent walls, pushing particles to the channel centerline. On the other hand, the shear gradient lift force is generated by the parabolic velocity curvature of Poiseuille fluid, driving particles away from the centerline of the microchannel.

The inertial microfluidics typically works in an intermediate *Re* range. And the phenomenon of particle migration was observed in various kinds of microchannels [29,30,31]. We have known that inertial lift force exerts on flowing particles, forming some equilibrium positions, and it closely depends on the geometry of the channel’s cross section. Ignoring the far weaker hydrodynamic forces (such as the Saffman force, Magnus force, etc.), the inertial lift force is mainly composed of the shear gradient lift force and the wall lift force. The analytical expression of the inertial lift force ($F_{L}$) exerting on a small rigid sphere particle (a/H<< 1) was, through the method of matched asymptotic expansions, first derived by Asmolov as: [32]

(1) $$F_{L}=f_{L}\rho_{f}\gamma^{2}a^{4}$$

This expression can be simplified as:

(2) $$F_{L}=f_{L}\rho_{f}U^{2}a^{4}/H^{2}$$

(3) $$f_{L}\propto H^{2}/a^{2}\sqrt{Re}$$

where γ is the shear gradient. The lift coefficient $f_{L}$ is a function of the lateral position of particles x and the Re [32,33]. The scaling was derived from experimental results, and it is found that $f_{L}$ remains nearly constant (0.5) when *Re* is less than 100 [23,31].

Meanwhile, the viscous drag force on a flowing particle is influenced by particle Reynolds number (*Re’ = ν_t_ρ_f_a/μ;* here *v_t_* is the relative velocity of the fluid to the particle). At the low *Re’*, the drag force exerting on a rigid spherical particle can be expressed as follows:

When the *Re’* is in the range between 10^−4^ to 0.2,

(4) $$F_{D}=3\pi r\mu \left(v_{f}-v_{p}\right)$$

When the *Re’* is in the range between 0.2 to 10^3^,

(5) $$F_{D}=3\pi r\mu \left(v_{f}-v_{p}\right)\left(1+0.15R{e^{\prime }}^{0.687}\right)$$

where r is the radius of the particle, $v_{f}$ is the velocity of the fluid, and $v_{p}$ is the velocity of the particle [34].

## 3. Spiral Microchannel

**Figure 2:**
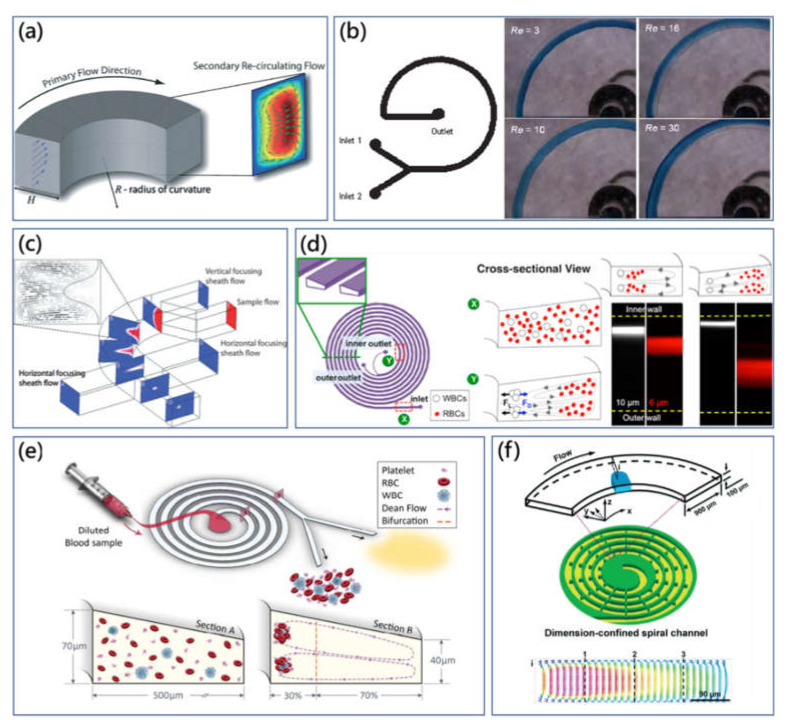
(**a**) Transverse counter-rotating microvortices within the cross section of the curved channel. Dean flow is generated by the inertial mismatch and centrifugation effects in the cross section of microchannel [4]. Adapted with permission from Di Carlo. (**b**) Optical microscopic images of the dyed fluid flow distribution captured at the end of the spiral micromixer, illustrating the mixing performance at different flow rates. *Re* is gradually increased from 3 to 30 [38]. Adapted with permission from Howell Jr. et al. (**c**) Schematic drawing of the three-dimensional focusing of co-flow streams in the planar spiral microchannel with the assistance of Dean vortices [39]. Adapted with permission from Mao et al. (**d**) Modified Dean flow separation method using the spiral microchannel with trapezoid cross section [40]. Adapted with permission from Wu et al. (**e**) High throughput blood plasma extraction using multiplexing slanted spiral microchannel with the trapezoidal cross section [27]. Adapted with permission from Rafeie et al. (**f**) Confined spiral microchannel with ordered micro-obstacles for the accelerated Dean flow particle manipulation [41]. Adapted with permission from Shen et al.

In the curved channel, the transverse secondary flow is generated within the cross sections due to the velocity mismatch. In Poiseuille flow, the fluid element near the centre area of the cross section possesses larger inertia, while the fluid element at the adjacent area of the channel sidewall possesses lower inertia. As a result, the fluid in the centre tends to migrate outwards, and the fluid originally located at the outer position moves laterally inwards through the top and bottom space based on the conserve mass principle [4], Figure 2a. The stable flow field within the cross section transforms into two symmetric counter-rotating microvortices, defined as Dean flow [35]. Several external parameters, including channel cross-sectional dimensions, Dean number (*κ*), *Re* and curvature radius of the channel can affect the magnitude and form of Dean flow. The dimensionless parameter *κ* was proposed by Berger et al. to evaluate Dean flow, expressed as [36]:

(6) $$\kappa ={\left(\frac{H}{2R}\right)}^{1/2}Re$$

where *R* is the radius of curvature.

Furthermore, the velocity of the Dean flow ($U_{D}$) and the Dean drag force ($F_{D}^{\prime }$) can be derived as [37]:

(7) $$U_{D}=1.8\times 10^{-4}De^{1.63}$$

(8) $$F_{D}^{\prime }=3\pi r\mu (U_{D}-v_{p})$$

Meanwhile, with the increase of Dean number, the center of the two symmetric vortices is going to move outwards to the outer channel wall in the radial direction [36].

The spiral microchannel is one of the widely used curved channels, which has been extensively investigated owing to its robust ability and easy principle [22,42]. As the curvature of the spiral channel is constant in a single direction, the generated Dean vortices at different curved parts along the spiral microchannel are approximately the same, which enables the prediction of the particle dynamics by the superposition of the inertial migration and Dean flow. Normally, flowing particles with similar density with the fluid experience coupled effects from the Dean flow drag force and inertial lift force, as the centrifugal force is negligible [17]. Di Carlo et al. introduced the inertial force ratio *R*_f_ (Dean flow drag force/inertial lift force) to evaluate the order of magnitude scaling between two effects determining the particle behavior [43]. It is suggested that the particle behaviour would be modified by the Dean drag force, as *R*_f_ was larger than 0.04 [44].

At the nascent stage, different fluids mixing was the pivotal problem for microfluidic development. The spiral microchannel was originally employed to generate Dean vertices performing the mixing functionality through increasing the interfacial area for diffusive mixing [38,45]. Jiang et al. presented that the periodic transverse Dean vortex patterns in curved microchannel enabled rapid chaotic mixing for Dean number over 140 [46]. Howell et al. proposed the Dean vortex-based micromixer using a spiral microchannel, as shown in Figure 2b [38]. The mixing effect was found to be improved as the applied *Re* increased from 1 to 10. Furthermore, the aspect ratio of microchannel influenced mixing performance as well. Low aspect ratio (0.5) strongly suppressed the vortex formation, while mixing could be enhanced as the ratio exceeded 1. Then, mixing performance using a spiral microchannel was investigated by Sudarsan et al. by introducing five spiral designs with different dimensions. In experiments with *Re* ranging from 0.02 to 18.6, mixing results were influenced by the flow rate, length of spiral contour and quantity of arc, while it has been demonstrated that the abrupt expansion change of the cross-sectional area was able to improve the mixing through the expansion vortex as well [45].

**Figure 3:**
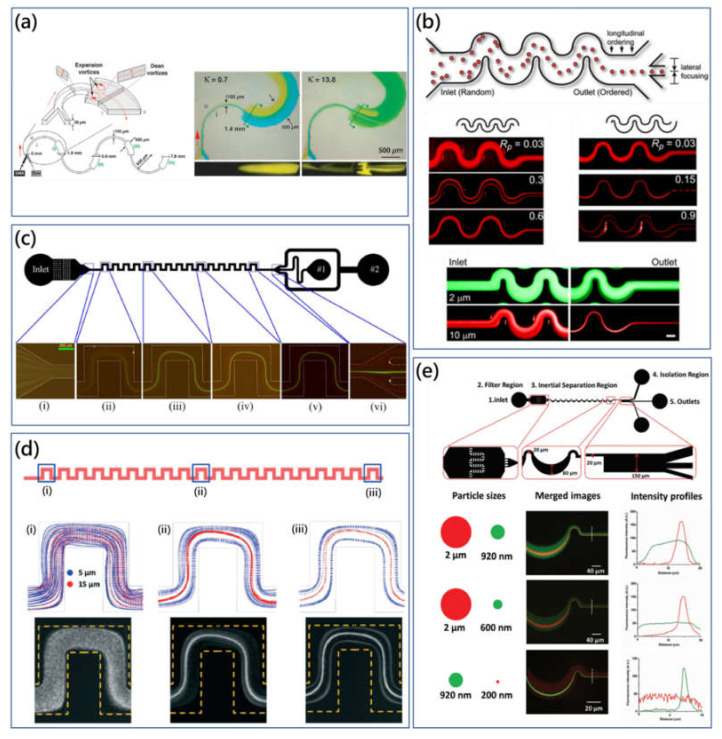
(**a**) Schematic diagram of the asymmetric serpentine micromixer (ASM) configuration and top microscopic view visualizing the color dye change in the expansion curved channel at different Dean numbers [24]. Adapted with permission from Sudarsan et al. (**b**) Fluorescent particle trajectories captured in the symmetric and asymmetric serpentine microchannel [23]. Adapted with permission from Di Carlo et al. (**c**) Continuous size-based particle separation using the symmetric serpentine microchannel. At moderated *Re*, different size particles receive corresponding Dean flow drag force, resulting in different focusing phenomena [61]. Adapted with permission from Zhang et al. (**d**) Consistent results of particle trajectories along the serpentine microchannel between experimental capturing images and simulation results [62]. Adapted with permission from Liu et al. (**e**) High-throughput micrometer and submicrometer size-based particle filtration approach using the customized asymmetric serpentine microchannel [63]. Adapted with permission from Wang et al.

Additionally, for confined Dean flow, the transverse Dean flow drag force has been demonstrated to contribute to ordered particle manipulations. The combination effects of Dean flow and inertial migration in the spiral microchannel was explored for the particle-focusing component of on-chip flow cytometry [39,47]. Mao et al. reported a three-dimensional hydrodynamic focusing in the planar spiral microchannel with the aid of one pair of sheath flows [39]. As shown in Figure 3c, Dean vortices transformed vertical co-flow streams into horizontal streams. The additional pair of sheath flows subsequently pinched horizontal streams forming the three-dimensional streamline focusing. Moreover, Bhagat et al. further developed the three-dimensional hydrodynamic focusing method in the spiral microchannel for a microscale flow cytometer [37]. As a powerful single-cell analysis tool, a commercial flow cytometer was normally bulky and expensive. The planar spiral microchannel design could take advantage of the Dean flow drag force and inertial lift force to focus the particles in three dimensions. With a laser-induced fluorescence module integrated, the on-chip flow cytometer system could offer a high detection throughput of 2100 particles/s. Moreover, using the adjusted curved spiral microchannel, Cruz et al. realized ultrasensitive submicro particle focusing [48]. Particles with size between 0.5 μm to 2.0 μm could be aligned on corresponding equilibrium positions at the end of the microchannel.

It is known that the direction of the streamline determines the particle migration tropism and the magnitude difference of drag force enables particle distributions at different positions, subsequently, particle separation can be realized. Yoon et al. investigated the size-based particle separation theory based on Dean vortex in the spiral microchannel [49]. The large particle with diameter larger than 0.72 times channel height *h* was guided by transverse flow in the center area to outward direction, while the small particle whose size was smaller than 0.27*h* was pushed by boundary circulating flow to the inward sidewall.

The spiral microchannel has been extensively exploited for applications of particle sorting and separation in past decades [37,47,50,51,52,53]. Evidence from the Equation (8), Dean flow drag force exerted on particle is dependent on the size of particles, which is useful for particle differentiation. Bhagat et al. successfully used a five-loop spiral microchannel to completely separate 7.32 μm and 1.9 μm particles at *Re* of 5 [54]. Inside the curved channel, small particles were retained in Dean flow, while large particles were equilibrated by the counterbalance of Dean flow effects and inertial effects to the new position. Recently, circulating tumor cells (CTCs) separation and enrichment have attracted great attention because of the important biomarker potential of CTCs in clinical diagnostics. Hou et al. developed a Dean Flow Fractionation (DFF) method through the spiral microchannel to continuously isolate CTCs from diluted blood samples [51]. Later, Sun et al. proposed the double-spiral microfluidic channel to continuously isolate tumor cells from blood sample [53]. The throughput of this on-chip method could be comparable with the conventional macroscale technology, reaching up to 2.5 × 10^8^ cells/min and the isolation efficiency could be more than 90%.

Meanwhile, the effect of the cross-sectional shape of the spiral microchannel on amplifying the difference of particle migration was investigated [40,55]. Various cross-sectional shapes such as trapezoid and pentagon were introduced for the spiral microchannel to control particle alignment using a specific Dean flow. Depicted as Figure 2d, Wu et al. proposed the novel spiral microchannel with a trapezoidal cross section to separate polymorphonuclear leukocytes (PMNs) and mononuclear leukocytes (MNLs) from diluted blood samples [40]. In the spiral microchannel with rectangular cross section, the large particle was focused on the inner sidewall by the Dean flow drag force, while the smaller particle was trapped near the core of Dean microvortices. The settling locations of different-size particles were overlapped, which reduced the separation efficiency. In contrast, the trapezoidal cross section shifted the core of microvortices to the outer sidewall with larger depth, for which the gap of different equilibrium positions was amplified and the broadening space at the outer side was able to accommodate more quantity of particles. Then, the particle-focusing mechanism within the spiral microchannel with the trapezoidal cross section was comprehensively investigated by Guan et al. [56]. Moreover, Warkiani et al. reported an ultra-fast, label-free CTCs-isolating inertial microfluidic device using the spiral microchannel with trapezoidal cross section [57]. The stronger Dean vortex trapped small size hematologic components while large CTCs were concentrated on another equilibrium position to the opposite direction. Compared with the previous method, the requirement of sheath flow is neglected, which improve the throughput by an order of magnitude. In the validation experiment, cancer cell line cells could be isolated from a 7.5 mL blood sample within 8 min for a high purity of over 80% (400–680 WBCs counts/mL, ~4 log depletion of WBCs). Furthermore, most recently, Rafeie et al. proposed the slanted spiral microchannel with trapezoidal cross section to isolate plasma in a high-throughput manner, Figure 2e [27]. In this scheme, the inner depth of the cross section was larger than the outer depth so that more locating space was prepared for blood cells deviated inwards. The proposed spiral device was able to isolate plasma with a purity of 100% and a throughput of 1.5 mL/min for diluted blood sample (0.5–1% Hct).

Additionally, the spiral microchannel was decorated with the confined microstructure to improve the expected manipulating performance on particles. Recently, Shen et al. presented the spiral microchannel with a library of ordered obstacles for establishing the sheathless, high-throughput, long-term and highly efficient particle-manipulation method [41]. As shown in Figure 2f, through the introduction of the micro-obstacles along one side of the spiral microchannel, cross-sectional area was decreased, therefore, the flowing streamline velocity within the microchannel was accelerated. The numerical simulation showed that the magnitude of transverse Dean flow was amplified. With application of the confined spiral microchannel, they successfully conducted the particle sorting for a ternary mixture, fluorescently labeled cell focusing and separation of CTCs from a complex blood sample and blood-plasma extraction. On the other hand, typical 2D planar spiral microchannel does not generate constant strong Dean flow. The Dean flow is greatly influenced by the varying curvature radius. As a result, spiral microchannels require the covering of a large footprint at the square-centimeter scale to realize particle manipulation. The compact 3D helical spiral microchannel can overcome this shortcoming by arraying the curved channel with a constant radius in a 3D microfluidic network [58,59]. Paie et al. presented that the tightly curved helical-spiral microchannel induced and maintained strong Dean flow to focus particles in three dimensions [59].

## 4. Serpentine Microchannel

Different from the spiral microchannel, and another typical curved channel, the curvature direction of the serpentine microchannel is not constant, which makes the Dean flow’s effects on particle migration much more complicated. It is challenging to predict the particle behavior through the coupled static superposition of inertial lift force and Dean flow drag force like that of the spiral channel. As a result, the research progress on the serpentine microchannel was a little slower compared with the spiral microchannel. However, due to its intrinsic advantages of small footprint and easy layout for structure optimization, the serpentine microchannel is still receiving increasing attention, and various interpretations of different particle-migration phenomena have been proposed by researchers [17].

Alternating fluid streamlines and fluid-element folding and stretching in the serpentine channel is advantageous for fluid mixing. The three-dimensional serpentine microchannel with C-shaped repeating units was reported by Liu et al. utilizing secondary flow to stretch and fold the fluids, increasing the interfacial area for mixing [60]. The serpentine microchannel provided 16 times the mixing performance compared with a straight microchannel. In this earliest work, the serpentine microchannel was a three-dimensional structure and fabricated by the complex double-sided KOH (Potassium Hydroxide) wet-etching technique. Afterwards, by harnessing the combination of Dean flow and horizontal expansion vortices, Sudarsan et al. presented an asymmetric serpentine micromixer (ASM) to improve microfluidic mixing performance through both curvature and width changing configuration [24]. As shown in Figure 3a, with *Re* ≈ 32, the ASM was able to achieve 80% mixing efficiency in the downstream distances, and the micromixing performance could be improved by increasing *Re*. In this configuration, optimization of the ASM was dependent on two geometric considerations: the aspect ratio of the cross section and expansion counterrotation angle.

The group of Di Carlo was the first to investigate hydrodynamic effects on particle focusing in serpentine microchannels [23,64]. Two types of serpentine microchannels were fabricated to investigate the influence of Dean flow on inertial migration, Figure 3b. In the symmetric serpentine microchannel, particles were observed to be focused at two side equilibrium positions firstly, and this focusing condition would be disturbed by increasing flow rate. On the other hand, in the asymmetric serpentine microchannel, particles were concentrated to the single streamline on the center of channel, and this equilibrium position would be blurred once again by increasing the flow rate. Hence, the relationship between inertial lift force and Dean flow drag force determined the quantity and location of particle equilibrium position within the serpentine microchannel. When inertial lift force dominates, particles could be observed to be concentrated under the modification of Dean flow drag. However, when Dean flow drag force dominates, particles would be mixed by the Dean flow microvortices. Furthermore, the asymmetric system induced the bias of particle distribution for the single equilibrium position. On the contrary, the system symmetry would generate transverse forwards and backwards particle movements for two symmetric equilibrium positions. Based on this fundamental research, Di Carlo et al. developed the rapid particle separation and filtration platform by differential inertial particle focusing at moderated Dean number using the asymmetric serpentine microchannel [64]. For the separation results, the purity of the small particle collected was more than 90%, though the small-particle recovery rate was relatively low, and the purity of filtrated large particles was also not ideal.

Meanwhile, our group has comprehensively investigated the mechanism of particle migration and differential focusing in symmetric serpentine microchannels and developed practical biological applications using the proposed mechanism [61,65,66,67]. Effects of particle centrifuge force on particle migration were studied [65]. In a low-aspect-ratio microchannel (0.2), mixing effects of Dean flow were weakened, while Dean flow microvortices were expected to benefit particle focusing. The centrifugal force of particles was important for particle focusing when the density difference between particle and fluid was significant. As shown in Figure 3c, the high-purity particle separation using the symmetric serpentine microchannel was proposed as the small particles were dominated by the inertial lift force while large particles were modified by Dean drag force [61]. The purity of particles were both over 90% at a high throughput of 600 μL/min. Recently, Zhang et al. systemically investigated effects of different parameters, including particle size, microchannel geometry, fluid viscosity and flow conditions on particle migration in the serpentine microchannel [67]. It was found that particle-blockage ratio, channel geometry, channel aspect ratio and Reynolds number could affect the particle-focusing performance significantly. Moreover, through the scaling parameter and analytical analysis, the single operational map for different particle-focusing patterns was derived, which could be useful for guiding the design of microchannels for particle separation. 

Additionally, numerical modeling has been introduced to calculate different forces in the inertial flow regime through the direct numerical simulation (DNS). However, it is not suitable for long microchannels with a complicated structure. Liu et al. presented a fitting formula for inertial microfluidics based on DNS data obtained in straight channel, which could be used to predict the particle trajectories in serpentine microchannels [62]. 

Due to the size limitation (a > 0.07H), the submicrometer or nanometer particle manipulation is always challenging to inertial microfluidics. However, bacteria, organelles and viruses whose sizes are smaller than 2 μm, are critically important for clinical diagnostics, environmental monitoring, food-safety testing, etc. [68,69]. By shrinking down the serpentine microchannel, Wang et al. presented the inertial focusing and separation method for submicrometer particles using the asymmetric serpentine microchannel [63]. This approach leveraged the coupled effects of Dean drag force and inertial lift force presented by Di Carlo mentioned above. The asymmetric serpentine microchannel showed a robust particle-focusing performance in a short distance within the large flow-rate range. Particles with a diameter of 2 μm could be focused from 100 to 1400 μL/min, while revealing a fantastic flow-rate-independence feature. Moreover, submicrometer particle separation was successfully achieved by filtrating the large particles, Figure 3e. At the flow rate of 80 μL/min, 2 μm particles could be filtrated from the 920 nm particles. 

Similar to spiral microchannels in 3D layout, researchers search for robust serpentine devices through modification with the novel physical module. Most recently, novel microfluidic systems were reported to employ customized serpentine configurations for biological applications. Martel et al. introduced the delicate design to combine particle focusing and microsiphoning for continuous bioparticle concentration [70]. The microdevice was an asymmetric serpentine microchannel integrated with a microsiphoning channel, by which cell-free fluid was siphoned, and cells could be concentrated by 400 times at a high throughput of 240 mL/h.

## 5. Microchannel with Expansion-Contraction Cavity Array (ECCA) or Obstructions

In addition to curved channels, microchannels with expansion-contraction cavity array (ECCA) are also employed in inertial microfluidics, taking advantage of geometry-induced secondary flow for particle manipulation. Compared with spiral or serpentine microchannels that make use of the velocity mismatch within cross sections, ECCA microchannels generate secondary flow due to the abrupt change of cross section in terms of size or shape. The secondary flow also consists of two counter-rotating microvortices similar to Dean flow, which was defined as Dean-like flow.

**Figure 4:**
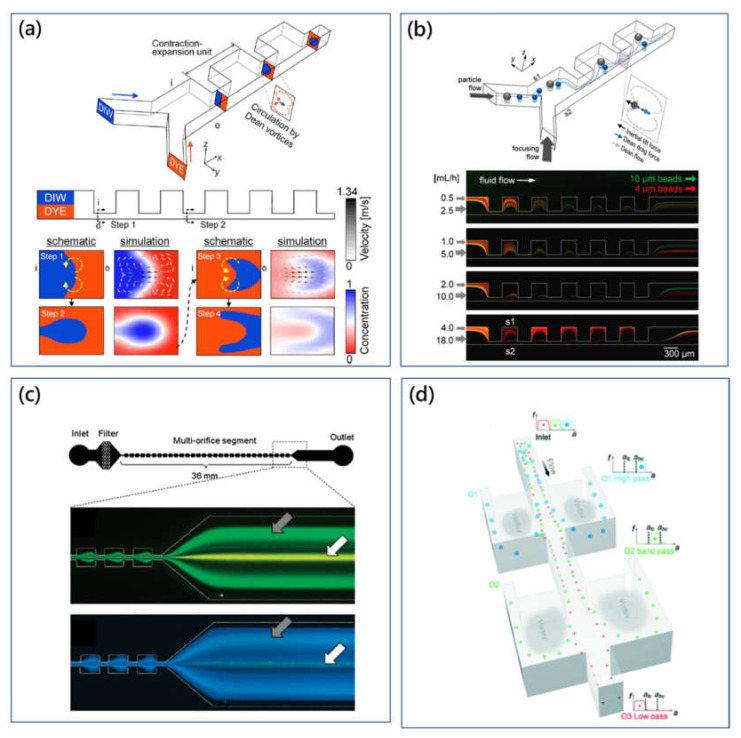
(**a**) Rapid laminating micromixer using the contraction-expansion array (CEA) microchannel and simulated Dean flow microvortices induced by centrifugation effects at the contraction channel [71]. Adapted with permission from Lee et al. (**b**) Schematic illustration of the large and small size particle migration within the CEA microchannel and the captured fluorescent particle (10 µm and 4 µm) trajectories with the increase of *Re* [73]. Adapted with permission from Lee et al. (**c**) Size-based multiorifice microfluidic particle separator using inertial lift force and Dean flow drag force [76]. Adapted with permission from Choi et al. (**d**) Schematic drawing of the size-based microfluidic multimodal particle sorting system with multiple pairs of expansion-contraction side collection chambers for the sorting of complicate components in the heterogeneous solutions [77]. Adapted with permission from Wang et al.

The group of Park has conducted comprehensive research on contraction-expansion array (CEA) microchannels for different particle manipulations [71,72,73,74,75]. In the CEA microchannel to mix two different fluid flows by the Dean microvortices [71], Figure 4a, Dean-like flow occurred at the contraction channel enabled continuous stretching and folding effects on fluid flows. This micromixer provided a 90% mixing performance when *Re* was from 4.3 to 28.6. Besides, with the assistance of a single sheath flow, the three-dimensional particle-focusing method using the same CEA microchannel was achieved [72]. The sheath flow with a high flow velocity gradually wrapped the low-velocity sample flow deviated by centrifuge-induced Dean-like flow, causing three-dimensional particle focusing. Moreover, the CEA microchannel has been also demonstrated to separate particles based on size difference [73]. In studies of focusing and mixing applications, the secondary flow was expected to be dominant on particle migration, while inertial lift force could be ignored because of the relatively low Reynolds number and small dimension. On the other hand, for particle separation, both inertial lift and Dean flow drag played an important role to guide particles to their corresponding equilibrium positions. As shown in Figure 4b, large particles were dominated by inertial lift force and concentrated to the equilibrium position near side S1, while small particles were dominated by Dean-like-flow drag force and concentrated to the equilibrium position near side S2. At the same time, this proposed sheath-flow-assisted inertial approach was available for the carrier-medium exchange, which was important for the biochemical assays. Furthermore, the CEA microchannel with sheath flow has been developed to separate blood cells from whole blood to achieve pure plasma as well as filtrate rare cancer cells from blood samples [74,75]. 

Meanwhile, in order to remove sheath flow to simplify the flow control, Park et al. developed the sheathless particle-manipulation method using the double-sided expansion-contraction cavity array microchannel [78]. The multiorifice microchannel could focus particles at two equilibrium positions on both sidewalls. Here, the particle equilibrium position was found to be affected by particle size and flow condition as well. Large particles were focused on the equilibrium position at the centerline, while small particles were located on the equilibrium positions on both sidewalls (Figure 4c) [76]. This size-based microfluidic particle-manipulation method was defined as multiorifice flow fractionation (MOFF). Furthermore, Sim et al. proposed a multistage multiorifice flow fractionation (MS-MOFF) device to improve the recovery rate and reduce the loss of purity through retreatments on particles [79]. The obtained recovery rate was found to increase from 73.2% to 88.7%.

Hur et al. presented a sheathless particle isolation method using an ECCA microchannel [80]. In principle, large target particles or cells could be isolated into the microscale cavity from heterogeneous solutions, and this reported microfluidic device was called “centrifuge-on-a-chip”. Particles were focused first in the high-aspect-ratio microchannel by inertial lift force. Then, in the expansion structure, the shear-gradient lift force pushed particles to the direction of expansion. Because the shear-gradient lift force was proportional to the size of the particle, large particles could be pushed deeper to the expansion cavity and trapped by the microvortices [81]. In massively parallel applications, capturing efficiency of the channel network was higher than 25%, as each cavity could trap 11 to 25 cells. 

The “centrifuge-on-a-chip” device can selectively trap, isolate rare cells based on size difference from background particles. And selecting sensitivity of this method was believed to be one of the highest for inertial methods [82]. Fundamentals of the manipulating mechanism and process were comprehensively investigated for isolating cancer cells from blood [81,83] and pleural fluids [84]. However, due to the limited capacity of the trapping cavity, the total number of the target cells obtained was normally no more than the level of hundreds. It was difficult to process a large volume sample. To solve the problem of limited capacity, Wang et al. presented the vortex-aided inertial microfluidic particle-separation method using the siphoning channel with symmetric expansion chambers on both sides [85]. The abrupt cross-sectional change generated vortices to siphon particles into the expansion chambers. Then, siphoned large particles were guided by the sheath flow within the chamber and released through the side outlets. Based on this vortex-assisted inertial microfluidic particle-isolation mechanism, the group of Papautsky developed the size-based microfluidic multimodal particle sorter using the high-aspect-ratio straight microchannel with multiple pairs of expansion-contraction side chambers, as shown in Figure 4d [77]. The cut-off size of each section in the microchannel could be modulated easily through change of flow rate and fluidic resistance of the channel network. Trinary particle mixtures could be individually separated by two steps in the microchannel with high resolution.

## 6. Multilayered Microchannel with Groove Array

Although the vast majority of microchannels’ cross section is restricted to regular rectangle due to characteristics of single-layer photolithography, multilayered microchannel can also be fabricated through the layer-by-layer stacking of multistep photolithography. Accordingly, some innovative works using straight multilayered microchannels with groove- or ridge-array structure onto the surfaces have been also explored by taking advantage of strong geometry-induced secondary flow to manipulate particles of interest. The secondary flow pattern is more complicated and unpredictable, which is distinct from the curvature-induced Dean flow discussed above [20].

**Figure 5:**
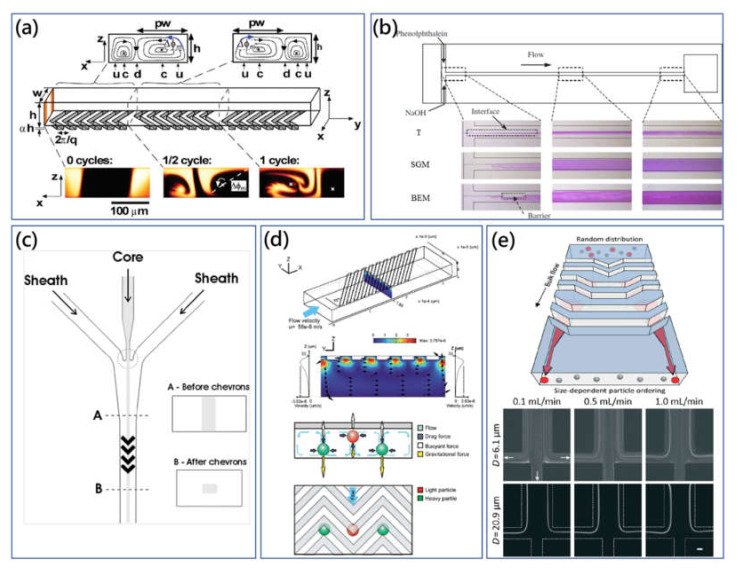
(**a**) Staggered herringbone mixer (SHM) using chaotic twisting flow by the slanted groove structure [86]. Adapted with permission from Stroock et al. (**b**) Optical images of the dyed fluid distribution in different microchannel configurations showing the mixing performance comparison between T-channel, slanted groove mixer (SGM) and barrier embedded mixer (BEM) [90]. Adapted with permission from Kim et al. (**c**) Layout of the herringbone-grooved microchannel used for multiwavelength microflow cytometer. Sample flow can be hydrodynamically concentrated and wrapped by the directed sheath flows with isolation from microchannel surfaces [91]. Adapted with permission from Golden et al. (**d**) Transverse circulating secondary flow microvortices induced by slanted groove structures on the top surface of microvortex manipulator (MVM) [89]. A particle’s equilibrium position is dependent on its density compared with the liquid medium. Adapted with permission from Hsu et al. (**e**) Flow-rate-insensitive continuous microfluidic filtration device with shifted groove structures on the top surface. The robust and simple microchannel can be interconnected with the syringe and operated manually through slow pushing [92]. Adapted with permission from Song et al.

Herringbone or ridge structure was first introduced to rapidly mix flowing fluids in a straight microchannel with the absence of turbulence and inertial effects at low Reynolds number (<1) (Stokes flow). Stroock et al. presented the pioneering microchannel with ridges placed on the top floor at the oblique angle of 45°, and they found that the transverse secondary vortices were generated due to the steady axial pressure gradient [86]. The generated twisting flow was also observed to be independent of *Re* in Stokes flow regime. As shown in Figure 5a, they successfully demonstrated that the staggered herringbone mixer (SHM) with ridged topography could realize effective microfluidic mixing at *Re* between 0 to 100. Then, the brief developing analysis and model of transverse pressure-driven secondary flow over ridge surfaces along a straight microchannel were conducted and derived based on the simple anisotropic effective boundary conditions to offer the practical workable guide of SHM mixer design [87,88]. Meanwhile, as it is found that transverse secondary-flow drag force was linearly proportional to *Re* (<2), the proposed microfluidic mixing technologies were typically insensitive to changing flow rate [89]. 

The addition of the SHM microfluidic mixer, slanted-groove mixer (SGM), barrier-embedded mixer (BEM) and other passive derivative mixers evolving from the original SHM have been developed [87,90,93,94]. In these works, Kim et al. have shown that the combination of periodically inserted barriers and an SGM T-channel could provide a better mixing performance, Figure 5b [90]. In BEM, the inserted barriers exerted spatial flow perturbation effects onto the secondary flow microvortices. Meanwhile, Sato et al. reported that slanted grooves on the top floor and both sidewalls in the three-dimensional microchannel could generate efficient short pitch spiral flow to improve chaotic mixing performance compared with the conventional SHM device [94]. Furthermore, Stott et al. proposed the herringbone-chip (HB-chip) utilizing geometry-induced secondary flow to enhance the mixing performance of blood samples [95].The interactions between target CTCs and coated antibodies were greatly increased through microvortices. In clinical experiments, the fabricated parallel HB-chip array showed the enhanced capture efficiency with the 26.3% improvement compared with the CTC-chip.

Beside microfluidic mixers, the multilayered microchannel with a groove pattern can be used to orderly manipulate the particle distribution based on their physical characteristics (such as particle size and density). In low *Re*, Stokes flow merely exerted a viscous drag on buoyant particles in the mainstream direction, and the magnitude of the viscous effects was relatively weak. In this case, some normally negligible effects like gravitational force are of more importance. Some creative studies employed these forces and geometry-generated secondary flow to focus or separate particles. Bernate et al. utilized the straight microchannel with an array of slanted open cavities on the bottom floor to realize the separation of suspended particles depending on the difference of size and settling velocity [96]. Particles were cumulatively deflected along different directions by the disturbed flow field in the vicinity and interior of the open cavity.

In the application of flow cytometry, Howell Jr et al. developed the microdevice with a set of grooves to wrap the sheath flow for the two-dimensional (2D) hydrodynamic sample focusing [97]. In this scheme, the sample flow was firstly confined horizontally by a single sheath flow, and grooves could subsequently deviate the single sheath flow to vertically wrap the concentrated sample flow in a 2D form. Here, with the aid of the groove-generated helical flow, complete particle focusing was realized without the requirement of the additional pair of sheath flows. Furthermore, the multiwavelength microflow cytometry with inserted optical fibers was developed using the herringbone-grooved microchannel [91]. As shown in Figure 5c, the herringbone grooves on the top and bottom disturbed the flow field and guided the sheath flow to sandwich sample flow in a single streamline. 

After that, Hsu et al. presented the microvortex manipulator (MVM) to passively focus particles and cells, and further developed the mechanism to separate binary particle solutions of different density particles (the difference could be as low as 0.1 g/cm^3^) in a parallel manner [89]. As shown in Figure 5d, the slanted groove structure on the top surface could deflect the fluid laterally along with the expansion groove structure. On the other hand, owing to the mass conservation principle, the fluid recirculated to the opposite direction at the bottom of the microchannel, forming a closed secondary flow vortex within the cross section. The combination of secondary flow drag force, buoyant force and gravitational force guided particles to new equilibrium positions, which are at interfaces of adjacent secondary flow vortices. For this technique, the amplitude ratio of the expansion ridge to the straight channel was a critical parameter for the generated secondary flow pattern. For example, in the microchannel of SGM with the high ratio (>0.3), a secondary flow microvortex rose within the whole channel, while the microvortex merely occurred in the straight main channel in this work (0.1). 

Besides, the group of Choi and Park introduced the novel concept of hydrophoresis [98]. In the hydrophoresis principle, groove pattern induced pressure-driven secondary flow, guiding particle movements, and the steric interaction between particles and channel’s walls retained particles into the disturbed transverse flow. For effective hydrophoretic focusing, the particle’s diameter should be larger than a half of the microchannel height, and the open cavity spacing should be larger than the particle size to prevent clogging, but not too large or the particles would be trapped into the groove structures. The continuous hydrophoretic filtration method was proposed using the straight microchannel with both slanted groove patterns and filtration obstacle patterns on the top and bottom surfaces [15]. For isolation of WBCs from RBCs, slanted grooves contributed to hydrophoretic focusing on both cells, while filtration obstacles filtrated large WBCs and withheld RBCs in the focusing streamline.

**Figure 6:**
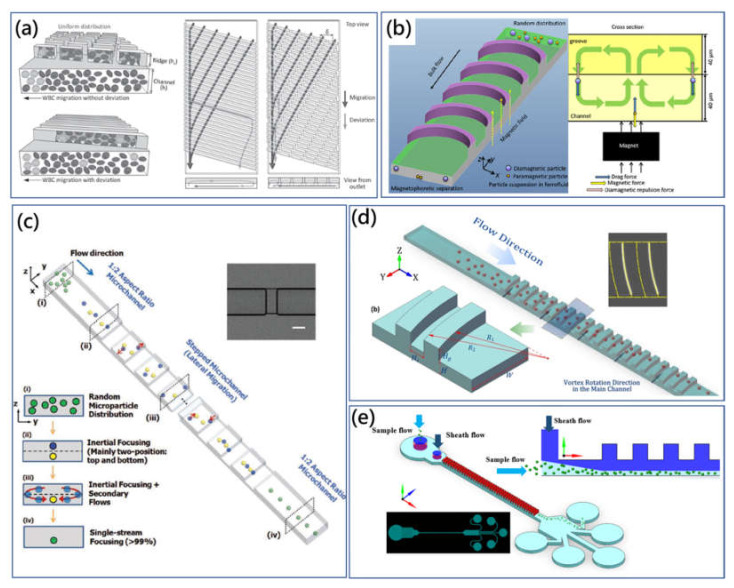
(**a**) Lateral deterministic migration-based WBC separation using the low aspect ratio straight microchannel with discontinuous slant array (DSA) [100]. Adapted with permission from Kim et al. (**b**) High-throughput, sheathless, magnetophoretic sorting of magnetic and nonmagnetic particles using the grooved microchannel [101]. Adapted with permission from Yan et al. (**c**) Three-dimensional, sheathless and high-throughput particle single-streamline focusing method using the stepped microchannel [102]. Adapted with permission from Chung et al. (**d**) Sheathless, high-throughput and three-dimensional particle-focusing method using a double-layered microfluidic device consisting of the low aspect ratio straight channel and arc-shaped groove array pattern on the top surface [103]. Adapted with permission from Zhao et al. (**e**) Enhanced sheath flow-assisted particle-focusing and separation method eliminating size limitation of conventional design [104]. Adapted with permission from Zhao et al.

Additionally, hydrophoresis requires precise control of a low flow rate. To address this issue, the flow-rate-insensitive microfluidic syringe filter was proposed for cell synchronization, Figure 5e [92]. There was the array of changing grooves on the top surface of microchannel. The shape of the groove was gradually shifted from centre to side, in which generated secondary flow guided large size particles to migrate laterally to the sides of the microchannel, regardless of flow-rate variation. As small particles could not meet the criterion of hydrophoretic focusing, they were randomly distributed. Moreover, Kim et al. reported the smart pipette with the designed microfluidic tip to separate plasma from whole blood sample using a similar strategy [99]. Later, Kim et al. revised their design of smart microfluidic pipette tip and presented the microchannel with discontinuous slanted groove array to preserve rare WBCs from blood cell populations [100]. As shown in Figure 6a, the WBC margination which resulted from the deformability and biconcave disk shape of RBCs contributed to lateral deterministic migration of WBCs. And the modified discontinuous groove array was used to solve the annoying deviation problem of WBCs to improve WBC enrichment. 

Hybrid microfluidic technologies which combine two or more physical principles have emerged recently. Our group proposed that the assistance of the active functional field (e.g., electric field and magnetic field) could improve the manipulation performance of hydrophoretic microfluidic devices to weaken the limitation of particle size [105,106]. Firstly, a dielectrophoresis-active hydrophoretic system was introduced to extract the plasma from the diluted blood sample in a relatively high throughput manner [107]. Because the relatively low working condition of hydrophoretic devices compared with other typical passive hydrodynamic means enabled enough retention time for particles under the electrical field, the dielectrophoresis would exert negative DEP force on the travelling particles and pushed them to the open cavity adjacent area where the particles under the size criterion could be concentrated by the steric helical flow successfully. Furthermore, the application of rapid magnetism-based continuous particle sorting was developed based on the improved hybrid hydrophoretic particle-manipulation approach [101]. As shown in Figure 6b, for the binary mixture of magnetic and nonmagnetic particles, the positive and active magnetophoretic force pulled the magnetic particle to the bottom and pushed the nonmagnetic particles away to the groove structures, forming the different equilibrium positions. At the flow rate of ~100 μL/min, the 6 μm magnetic particles were enriched at a purity of ~85%, and the focusing efficiency could be increased to over 95%.

The studies mentioned above are mostly under the regime of Stokes flow with low *Re*, where fluid inertia is negligible. A particle’s motion is dominated by the viscous drag force from the geometry-induced secondary flow. However, inertial effects cannot be neglected with the increase of *Re* in the microchannel. Song et al. illustrated that the inertial effects of the microchannel on the high *Re* could change the original hydrophoretic particle migration regime [108]. And they demonstrated a multifunctional microfluidic technique to focus and separate particles through adjusting the balance between the hydrophoresis and inertial effects. Recently, Mao et al. implemented numerical simulations to investigate the particle migration in the microchannel with diagonal grooves on both the bottom and top surfaces at a high *Re* (~20) [109]. The inertial effects of the low-aspect-ratio channel concentrated the particles to the long sidewalls. As the vertical component of the equilibrium positions for the large and small size particles were different, the directions of the drag force on large and small particles were opposite. In another work, Chung et al. employed a double-layered stepped microchannel which consisted of a low-aspect-ratio straight channel and the orthogonally located stepped-groove array on the top surface taking advantage of the geometry-induced secondary flow to modify the original multiple inertial particle equilibrium positions to a single-streamline [102], Figure 6c. The microchannel could work at *Re* of 83.33 to focus the 9.9 µm particles on the central equilibrium position promising a fascinating high throughput of 36,000 particles/s. They investigated the induced secondary flow at Stokes flow (*Re* = 0.2778) and laminar inertial flow (*Re* = 83.33) for the comparison study using numerical simulation. It is shown that the secondary flow at *Re* ≈ 0 was negligible with the tiny lateral fluid flow displacement, while the magnitude of the displacement was significantly amplified with *Re* = 83.33. 

Our group investigated the high-throughput and sheathless passive microfluidic particle focusing using geometry-induced secondary flow at high *Re* in the microchannel with arc-shaped groove arrays [103]. Inertial lift force was used to balance with secondary flow drag force at new equilibrium positions. As shown in Figure 6d, through the counterbalance between inertial lift force and secondary flow drag force, the inertial microfluidic method showed good three-dimensional focusing on different size particles as well as biological cells (such as Jurkat cells) in a high-flow rate of over 1000 µL/min. Furthermore, the flow-rate-insensitive particle filtration method was also developed based on a similar principle [110]. In the double-layered microchannel with grooves, secondary flow microvortices induced by the pressure gradient varied in both magnitude and shape with increasing *Re*, and consequently brought about different modes of particle focusing [111]. Moreover, we found that the selective utility of geometry-induced secondary flow could improve the manipulation efficiency [104]. As shown in Figure 6e, the single top sheath flow was employed in particle manipulation to overcome the limitation of focusing on small particles (2 μm to 5 μm). The introduction of top sheath flow pushed particles to the bottom region avoiding the deviation of geometry-induced secondary flow and to drive target particles to the equilibrium position more efficiently. Moreover, the sheath flow did not require a precise inlet flow rate control and this proposed inertial method was able to manipulate particles under a wide range of flow rate ratios. 

## 7. Discussion and Conclusions

In this review, we comprehensively discussed the phenomenon, theory and applications of secondary flow in inertial microfluidics. In a straight microchannel, particle-focusing positions are greatly dependent on the microchannel dimensions, particle and fluid physical characteristics. Introduction of secondary flow can enable the particle of interest to switch to the new position. Based on the hydrodynamic principle, many passive inertial microfluidic methods were proposed. According to the type of the channel structure, they are mainly classified as five parts discussed in this review, including spiral, serpentine, channel with ECCA, channel with obstructions and multilayered microchannel with groove array. The secondary flow can be finely tuned by the customized microchannel and possesses a great particle-manipulation ability with some useful features like flow-rate insensitivity.

The secondary flow is a dispensable component in inertial microfluidics. It transforms the multiple focusing positions into a single equilibrium position, and the particle-focusing positions can be also modified precisely by the pattern and magnitude of secondary flow, which enables a variety of microfluidic functions on particle and fluid manipulation. Based on it, a variety of biomedical applications using secondary flow in microfluidics have been demonstrated, such as blood plasma extraction [27], CTC isolation [57] and flow cytometry [37], etc. Compared with other microfluidic technology, where the inertial effect is nearly negligible at a low flow speed in the microchannel. The effects of fluid inertia should be considered when the flow Reynolds number is ~10. Besides, secondary flow can significantly improve the functionality of inertial physics for different applications.

Although it has achieved significant progress on the investigation of physical fundamentals, development of manipulation techniques and biomedical applications, some limitations need to be overcome to promote the performance of secondary flow in inertial microfluidic technology. For example, Dean flow can be quantitatively analyzed through the Dean number in the aspects of magnitude and vortex pattern [36]. However, complicated secondary flow generated by the double-layered microchannel with topology pattern is not consistent with the established regulation of Dean flow. Different patterns of secondary flow have been observed including single ellipse shape vortex, asymmetric double vortices and Dean-like vortices [89,111]. Through experimental studies, parameters of cross-sectional aspect ratio, groove- or ridge-pattern style, amplitude ratio between two layers’ heights and flow rate have shown effects on the geometry-induced secondary flow. Although some empirical conclusions are summarized for a rough scheme and design of double-layered microchannel, systematic quantitative analysis of secondary flow for precise particle manipulation is still required.

For biological applications, body fluid samples (e.g., DNA, saliva, blood plasma, etc.) exhibit nonnegligible non-Newtonian characteristics, as fluid viscosity is not constant depending on the shear gradient (shear-thinning) [112,113,114]. Despite the assumption that diluted samples are treated as Newtonian fluids, the viscoelastic effects might still be always obvious and affect the performance of the inertial microfluidics. On the other hand, by substituting the Newtonian fluid with the non-Newtonian fluid (e.g., polyethylene oxide (PEO) solutions and DNA solutions), elastoinertial particle migration can be achieved, taking advantage of both elasticity and inertia [115,116]. In the new regime of non-Newtonian physics, besides inertial lift force and secondary flow drag force, elastic force should also be considered. Furthermore, it is shown that, leveraging the extra introduction of elastic force, particle viscoelastic migration could be further customized for more precise manipulations [19].

Moreover, hybrid microfluidic platforms which combine two or more principles are proposed to bring more flexibility and functionality of microfluidic systems [117,118]. Inertial microfluidics have been demonstrated to integrate with sheath flow optophoresis, dielectrophoresis, etc. to manipulate target particles at high sensitivity [71,119,120,121,122]. Moreover, oscillatory inertial microfluidics can focus smaller-size particles with a small footprint microchannel [123,124,125,126]. Compared with the inertial microfluidics flowing in a constant direction along a pressure gradient, oscillatory flow can switch the flow direction at the high frequency, resulting in the practical infinite microchannel effects. The sufficiently long flowing distance enables a longer time of transverse inertial effects for functionality. It is expected that this novel mechanism can be also implemented in curved microchannels or microchannels with obstacles. The secondary flow direction can be switched with the mainstream direction, this may lead to different particle-focusing patterns compared to that in a constant flow direction, which needs a systematic study. Furthermore, for fluid mixing, switching flow direction alternatively using oscillatory flow, the fluid passing path increases and the stretching and folding frequency is greatly enhanced, which could be beneficial for a better mixing performance.
